# A microrna screen to identify regulators of peritoneal fibrosis in a rat model of peritoneal dialysis

**DOI:** 10.1186/s12882-015-0039-z

**Published:** 2015-04-09

**Authors:** Fan Lin, Xu Wu, Huidi Zhang, Xiaohan You, Zhoucang Zhang, Rongrong Shao, Chaoxing Huang

**Affiliations:** Department of Nephrology, The First Affiliated Hospital of Wenzhou Medical University, 2 Fuxuexiang Street, Wenzhou, Zhejiang 325000 China

**Keywords:** Peritoneal fibrosis, miRNA, Peritoneal dialysis, Epithelial-to-mesenchymal transition

## Abstract

**Background:**

Peritoneal fibrosis is a common complication in patients treated with long-term peritoneal dialysis. The aim of this study was to identify the microRNAs (miRNAs) involved in regulation of peritoneal fibrosis in a rat model of peritoneal dialysis.

**Methods:**

Twenty-four Sprague–Dawley (SD) rats were randomly allocated into three groups: (i) Control group (Cg, n = 8); (ii) Saline group (Sg, n = 8): daily intraperitoneal injection with 0.9% normal saline; (iii) Hypertonic dialysate group (HDg, n = 8): daily intraperitoneal injection with 4.25% peritoneal dialysis solution. Rats were sacrificed after four weeks for histological evaluation of peritoneal membrane and the expression of α-SMA and COL-1. A miRNA screen was performed using microarray analysis to identify differentially expressed miRNAs, which were then validated by real-time PCR.

**Results:**

Compared with the control and the saline groups, hypertonic dialysate group showed impaired peritoneal function accompanied by a spectrum of morphological changes including thicker peritoneal membrane, higher collagen deposition, infiltration of mononuclear cells and neovascularization in the peritoneum. Increased mRNA and protein levels of α-SMA and COL-1 were observed in hypertonic dialysate group, indicating the progression of peritoneal fibrosis. The miRNA screen identified 8 significantly down-regulated miRNAs (miR-31, miR-93, miR-100, miR-152, miR-497, miR-192, miR-194 and miR-200b) and one highly up-regulated miRNA (miR-122) in the hypertonic dialysate group. The results were confirmed by real-time PCR.

**Conclusions:**

Altered miRNA expression in peritoneum was found in the rat model of peritoneal fibrosis, indicating that these miRNAs may be associated with pathogenesis of peritoneal fibrosis.

**Electronic supplementary material:**

The online version of this article (doi:10.1186/s12882-015-0039-z) contains supplementary material, which is available to authorized users.

## Background

Peritoneal dialysis (PD) has been adopted worldwide as a renal replacement treatment for patients with chronic kidney disease. It has been used as an alternative to hemodialysis due to its advantages in controlling blood pressure and maintaining electrolyte balance [1,2]. In addition, PD does not require routine visits to a hospital or medical facility and thus offers a better quality of life and lower cost to the patients [3,4]. However, long-term PD is usually associated with development of structural and functional alterations in the peritoneal membrane [5,6]. Several risk factors for development of peritoneal fibrosis in PD patients have been implicated, including the bioincompatibility of hyperglycemic PD solutions, recurrent peritonitis, and increased concentration of growth factors such as FGF-2, TGF-β, CTGF, VEGF, etc. [7,8]. These factors may result in changes in the morphology of the peritoneal mesothelial cells and sediment of the extracellular matrix in peritoneum, and those changes ultimately lead to peritoneal fibrosis and ultrafiltration failure. Therefore, prevention and treatment of peritoneal fibrosis are critical to maintain the peritoneal membrane integrity and prolong PD treatment in patients. Recent studies have demonstrated that the anti-fibrogenic strategies, such as development of a more biocompatible PD solution, treatment of TGF-β-inhibiting proteoglycan and inhibition of the renin-angiotensin system, have the potential to attenuate or prevent the onset of peritoneal fibrosis in multiple animal models [9-12].

miRNAs are 19 to 25-nucleotide, non-coding RNA transcripts, and thought to be instrumental in controlling eukaryotic cell functions by modulating the post-transcriptional activity of target mRNAs through translational repression or mRNA degradation [13,14]. Because of the sophisticated roles miRNAs play in regulating various basic cellular processes such as proliferation, differentiation and apoptosis, dysregulation of miRNAs has been associated with a wide spectrum of human malignancies [15,16]. Recently, the involvement of miRNAs in different fibrotic disorders, including systemic sclerosis, liver cirrhosis, cardiac fibrosis and idiopathic pulmonary fibrosis, has attracted a considerable amount of attention [17-22]. There is accumulating evidence that shows the miRNA-regulated epithelial-to-mesenchymal transition (EMT) in human peritoneal mesothelial cells, which has been indicated by previous research as a key mechanism for the development and progression of peritoneal fibrosis [23-25]. Furthermore, recent studies have discovered the enrichment of specific extracellular miRNAs in peritoneal dialysis effluent, implying the potential of those miRNAs as diagnostic biomarkers [26-28]. More direct evidence comes from a study using a mouse model of peritoneal dialysis, in which microRNA-29 (miR-29) was identified to be an inhibitor of peritoneal fibrosis through suppression of TGF-β signaling pathway [29]. Therefore, it will be great interest to perform a comprehensive miRNA screen to identify additional regulatory candidates and examine their roles in pathogenesis of peritoneal fibrosis.

In this study, we performed an extensive screen to identify miRNAs with potential regulatory function in peritoneal fibrosis using a rat PD model. This study set the foundation for further exploration of the miRNA machinery in peritoneal fibrosis and for development of targeted therapy in PD patients.

## Methods

### Animals

Male SD rats weighing 140 to 160 g were purchased from SLAC Laboratory Animals Co (Shanghai, China). They were allowed to acclimatize for 1 week before the beginning of the experiment. Animals were maintained under specific pathogen free (SPF) conditions and allowed free access to food and water. All animal experiments in this study were approved by the Animal Care Committee of the Wenzhou Medical College of China (SCXK2007-0005) and conducted in accordance with “Recommendations on the Establishment of Animal Experimental Guidelines” approved at the 80th General Assembly of the Japanese Science Council in 1980.

Twenty-four SD rats were randomly divided into three groups: (i) Control group (Cg, n = 8); (ii) Saline group (Sg, n = 8): daily intraperitoneal injection with 0.9% normal saline; (iii) Hypertonic dialysate group (HDg, n = 8): daily intraperitoneal injection with 4.25% peritoneal dialysis solution (Baxter HealthCare, Deerfield, IL, USA). The volume of saline and 4.25% peritoneal dialysis solutions used in daily injection was 20 ml. The duration of treatment was four weeks for establishment of peritoneal fibrosis, which was determined by earlier studies and our previous observation [30,31].

### Peritoneal function test and sample collection

After four weeks of treatment, a 2-hour peritoneal equilibration test (PET) was performed. During each PET, rats were given 20 ml of 4.25% glucose peritoneal dialysis solution (PDS) via a 20-gauge needle into the peritoneal cavity. Dialysate samples (1 ml) were taken at 2 hours after infusion. Blood samples were taken from the inferior vena cava at the end of the PET. During the PET, animals were awake and had free access to water and food.

The dialysate and blood samples were centrifuged and stored at −20°C until assayed. Concentrations of creatinine and glucose in serum and dialysate were measured by enzymatic methods using a Hitachi 7600–110 Automated Biochemistry analyzer (Hitachi, Tokyo, Japan). Net ultrafiltration (UF) was determined as the volume of fluid removed after the 2-hour PET subtracted by the volume of fluid administered. The efficiency of solute transport across the peritoneal membrane was determined by glucose reabsorption (D_2_/D_0_, concentration of glucose in dialysate at 2 hours relative to that in the infused dialysis solution) and creatinine transport (D/P_urea_, dialysate to plasma concentration ratio of creatinine at 2 hours).

Following sample collection, all rats were sacrificed by bleeding from the abdomen. The peritoneal cavity was opened with minimal handling or trauma, and tissues of the visceral and parietal peritoneum were taken for further measurement.

### Cytologic examination and histology

White blood cell count in peritoneal effluent was obtained using an Olympus CX31 microscope (Olympus, Tokyo, Japan).

Each peritoneum was fixed with 4% paraformaldehyde solution and processed for paraffin embedding. Tissue sections (4 μm) were then stained with hematoxylin and eosin and Masson’s trichrome stain for histological examination of peritoneal mesothelial cells, thickness of the peritoneal tissues, inflammatory cell infiltration, and small vascular proliferation using an Olympus CX31 microscope.

### Immunohistochemical staining of the peritoneum

For immunohistochemistry (IHC), tissue sections were deparaffinized, rehydrated, and incubated with 3% hydrogen peroxide to inhibit the activity of endogenous peroxidase. The sections were then incubated with primary antibodies against α-SMA and COL-1 (Abcam, Cambridge, MA, USA) overnight at 4°C. On the next day, the sections were incubated with biotin-conjugated secondary antibodies, followed by incubation with horseradish peroxidase (HRP)-conjugated streptavidin. The resultant primary-secondary antibodies-biotin-streptavidin-HPR complex was then stained with diaminobenzidine (DAB), which led to reddish-brown stains representing positive immunoreactions. The reaction was interrupted by rinsing with distilled water. Samples were then contrasted with Mayer hematoxylin and examined using an optic photomicroscope at a magnification of 400X. To include a negative control, phosphate buffered saline (PBS) was used in place of the primary antibodies. For each tissue section, 10 different images were randomly selected and analyzed by semi-quantitative assessment using the Image-Pro Plus 6.0 software (Media Cybernetics, Inc., Rockville, MD, USA). The mean optical density was calculated by the overall optical density from all positive staining divided by total area.

### RNA extraction and quality determination

Total RNA was extracted from the peritoneum of rats using the Trizol Reagent according to the manufacturer’s instructions (Invitrogen, Grand Island, NY, USA). The RNA concentration and purity were determined using a Varioskan Flash Multimode Reader (Thermo Scientific, Waltham, MA, USA). The concentration of total RNA (μg/ml) was calculated using the formula: OD_260_ X dilution factor X 40 μg/ml. An OD_260/280_ ratio between 1.9 and 2.1 was considered an indication of high quality RNA, thereby making the RNA sample suitable for gene expression analysis. RNA integrity was also assessed using the Agilent 2100 Bioanalyzer (Agilent Technologies, Santa Clara, CA, USA), which detected the integrity of 18S and 28S RNA peaks.

### Microarray analysis

For each sample, 500 ng of total RNA was labeled using the FlashTag Biotin HSR RNA Labeling Kit and prepared for the GeneChip miRNA Arrays according to the manufacturer’s recommendations (Genisphere, Hartfield, PA, USA).

After labeling, samples were hybridized on the Affymetrix GeneChip miRNA 2.0 Arrays (Affymetrix, Santa Clara, CA, USA) in a volume of 100 μl reaction mix at 48°C and 60 rpm for 16 hours. Immediately following hybridization, the arrays were washed and stained with streptavidin-phycoerythrin conjugates in the GeneChip Fluidics Station 450. Finally, the arrays were scanned using a GeneChip Scanner 3000 7G (Affymetrix).

The microarray data were analyzed using the Affymetrix GeneChip Command Console (AGCC) software. Fold changes higher than 2 or lower than 0.5 were considered to be significantly different between two groups.

### Real-time PCR analysis

RNA expression in visceral peritoneum was evaluated by real-time PCR analysis. cDNA was synthesized from the purified total RNA using a cDNA Reverse Transcription Kit (Thermo Scientific) according to the manufacturer’s instructions. cDNA was amplified using the SYBR Green PCR Master mix in a 7500 Real-Time System (Applied Biosystems, Grand Island, NY, USA). Relative expressions of mRNA and miRNA were calculated using the 7500 Real-Time System software.

For miRNA, the real-time PCR reaction was maintained at 95°C for 3 min, followed by 95°C for 5 s and 60°C for 35 s for 40 cycles. For mRNA, the real-time PCR reaction was maintained at 95°C for 3 min, followed by 95°C for 15 s and 60°C for 1 min for 40 cycles. The dissociation curves were analyzed to ensure amplification of a single PCR product. In order to ensure reliability of the results, all samples were processed in triplicate. Quantification was performed by the comparative cycle threshold (Ct) method [32]. To normalize the data, the GAPDH mRNA and the U6 miRNA were used as housekeeping genes. The sequences of PCR primers are listed in Additional files 1 and 2.

### Statistical analysis

Statistical analysis was performed using the SPSS 18 software (IBM, Armonk, New York, USA). All numerical results were expressed as the mean ± standard deviation (SD). Comparison between groups was made by one-way analysis of variance (ANOVA). Pairwise comparison was made by Tamhane’s T2 test. A value of *P* <0.05 was considered statistically significant.

## Results

### Hypertonic dialysate group had reduced ultrafiltration capacity

Net ultrafiltration (UF) was measured in a 2-hour PET after four weeks of dialysis treatment. Compared with the control group and the normal saline group, the hypertonic dialysate group showed significantly reduced UF capacity and glucose reabsorption (D_2_/D_0_) while increased dialysate-to-plasma urea ratio (D/P_urea_) (all *P* <0.05) (Table 1). These results indicated that the peritoneal function was greatly impaired following long-term peritoneal dialysis.

**Table 1:** **Peritoneal function test in three groups after four weeks treatment**

**Groups**	**N**	**UF (ml)**	**D**_**2**_ **/D**_**0**_	**D/P**_**urea**_
Control	8	27.97 ± 1.11	0.28 ± 0.04	0.12 ± 0.03
Saline	8	28.40 ± 0.95	0.27 ± 0.03	0.12 ± 0.05
Hypertonic dialysate	8	10.53 ± 0.75^*#^	0.14 ± 0.02^*#^	0.42 ± 0.05^*#^

^*^compared with the control group, *P* <0.05.

^#^compared with the saline group, *P* <0.05.

### Hypertonic dialysate group showed morphological changes of peritoneum

White blood cell count in peritoneal effluent was measured for signs of inflammation and peritonitis. In all three groups, the white blood cell count was lower than 100/mm^3^ when examined using an optic microscope, indicating that no rats in three groups developed peritonitis.

**Figure 1:**
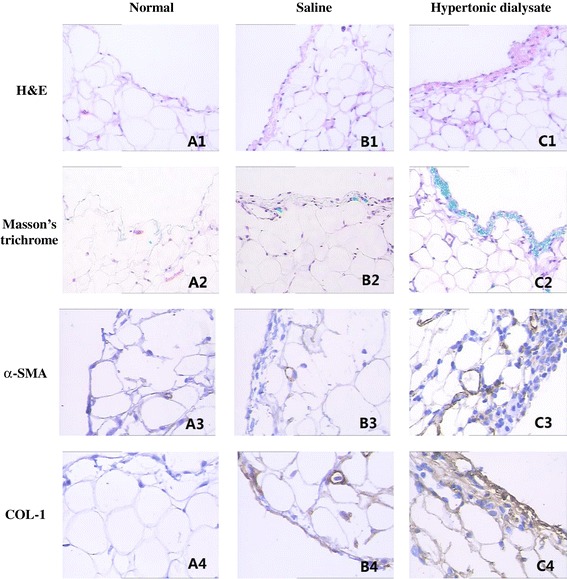
Histological examination of rat peritoneum and expression of α-SMA and COL-1 after four weeks treatment. **(A1, B1 and C1)** H&E staining (original magnification: X200) of peritoneum isolated from the control group **(A1)**, the normal saline group **(B1)**, and the hypertonic dialysate group **(C1).** **(A2, B2 and C2)** Masson’s trichrome staining (original magnification: X200) of parietal peritoneum isolated from the control group **(A2)**, the normal saline group **(B2)**, and the hypertonic dialysate group **(C2)**. Collagen was stained green. Note that the peritoneum from the hypertonic dialysate group had increased collagen deposition compared with the other two groups. **(A3, B3 and C3)** α-SMA staining (original magnification: X400) of peritoneum from the control group **(A3)**, the normal saline group **(B3)**, and the hypertonic dialysate group **(C3)**. **(A4, B4 and C4)** COL-1 staining (original magnification: X400) of peritoneum from the control group **(A4)**, the normal saline group **(B4)**, and the hypertonic dialysate group **(C4)**.

To assess the potential structural change induced by hypertonic dialysis in the peritoneal membrane, histological examination was performed in tissue samples of peritoneum obtained from the three groups. The peritoneal membrane in the control and saline groups appeared as a monolayer of mesothelial cells (Figure 1,A1 and B1). However, the peritoneum was thicker and appeared more edematous in the hypertonic dialysate group (Figure 1,C1). Mononuclear cell infiltration, fibroblastic activation, neovascularization and interstitial edema were more prominent in the hypertonic dialysate group than in the control and saline groups. The results from Masson’s trichrome staining showed that the collagen deposition was significantly higher and the peritoneal membrane was much thicker in the hypertonic dialysate group than in the control and saline groups (Figure 1,A2,B2 and C2).

### Hypertonic dialysate group had increased expression of EMT markers

**Figure 2:**
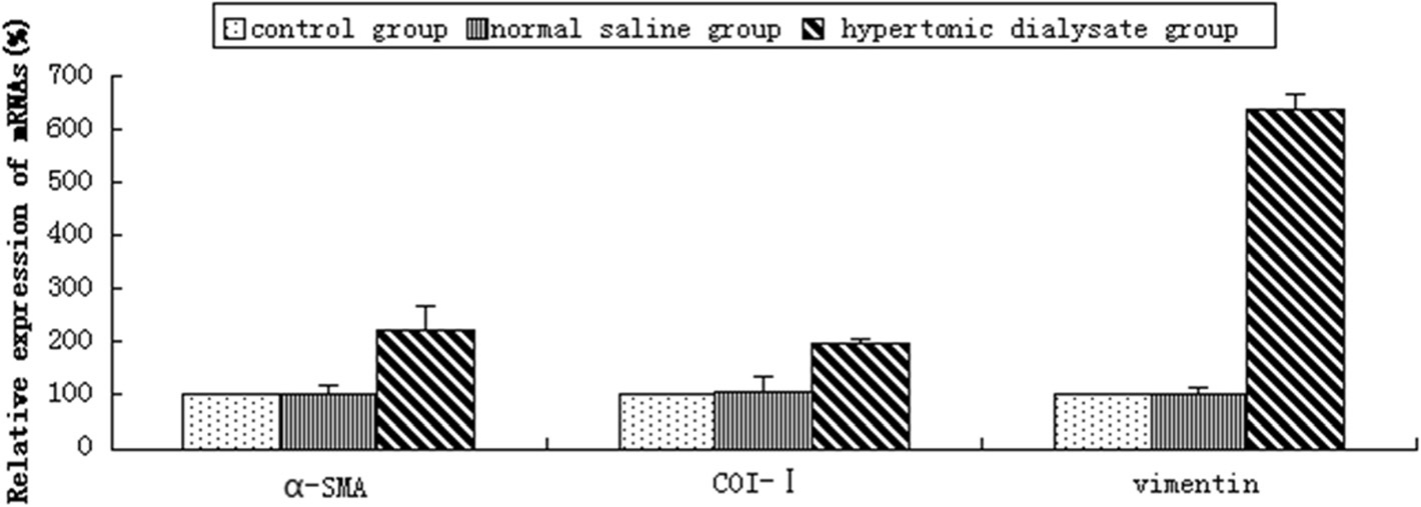
Relative expression of α-SMA, COL-1 and vimentin in visceral peritoneum after four weeks treatment. Relative mRNA expression (%) of α-SMA, COL-1 and vimentin was measured by real-time PCR analysis and comparisons were made between the control, normal saline and hypertonic dialysate groups. The results are presented as mean ± SD from 3 independent experiments. The differences in expression of α-SMA, COL-1 and vimentin between the saline group and the hypertonic dialysate group were significant (all *P* <0.05).

During peritoneal fibrosis, mesothelial cells acquire a fibroblast-like phenotype in a process called EMT, which is critical in driving changes in extracellular matrix. Alpha-smooth muscle actin (α-SMA) and type I collagen (COL-1) are mesenchymal markers, the expression of which corresponds to morphological changes in the peritoneum [33,34]. α-SMA (Figure 1,A3,B3 and C3) and COL-1 (Figure 1,A4,B4 and C4) were highly expressed in the peritoneal mesothelial cells, peritoneal mesothelium matrix, infiltrated inflammatory cells, and vascular endothelial cells in the hypertonic dialysate group. When using the semi-quantitative imaging software to analyze the staining intensity, the hypertonic dialysate group showed significantly higher expression of α-SMA and COL-1 compared with the control and saline groups (all *P* <0.05) (Table 2). No significant differences in expression of α-SMA and COL-1 between the control and saline groups were observed (all *P* >0.05). The results from IHC were further validated in mRNA level by real-time PCR. Consistently, α-SMA and COL-1 levels showed no significant differences between the control and saline groups (all *P* >0.05) (Figure 2). In contrast, expression of α-SMA and COL-1 were both up-regulated in the hypertonic dialysate group (all *P* <0.05) (Figure 2). Additional EMT marker vimentin [35] was also investigated, and highly up-regulated expression was observed in the hypertonic dialysate group (Figure 2). These results demonstrated that the progression of peritoneal fibrosis was accompanied by morphological changes and development of EMT in rat peritoneum treated with hypertonic dialysate.

**Table 2:** **Expression of α-SMA and COL-1 (measured by mean optical density) after four weeks treatment**

**Groups**	**α-SMA**	**COL-1**
Control	0.0222 ± 0.0114	0.0433 ± 0.0058
Saline	0.0454 ± 0.0090†	0.0509 ± 0.0015†
Hypertonic dialysate	0.1464 ± 0.0052^*#^	0.1379 ± 0.0037^*#^

^*^Compared with the control group, *P* <0.05.

^#^Compared with the saline group, *P* <0.05.

†Compared with the control group, *P* >0.05.

### Microarray analysis identified differentially expressed miRNAs

An extensive miRNA screen was performed to identify miRNAs which were differentially regulated during the process of peritoneal fibrosis. The results were analyzed by making comparisons between the hypertonic dialysate and saline groups, between the hypertonic dialysate and control groups, and between the saline and control groups. It was found that 8 miRNAs were significantly and consistently down-regulated in the hypertonic dialysate group (miR-31, miR-93, miR-100, miR-152, miR-497, miR-192, miR-194 and miR-200b), and within which miR-192, miR-194 and miR-200b were also down-regulated in the normal saline group (Table 3). Significantly increased expression of miR-122 was observed in the hypertonic dialysate group compared with the saline and control groups (Table 3).

**Table 3:** **Change of microRNA expression by microarray analysis in three groups after four weeks treatment**

	**HDg vs. Sg**	**HDg vs. Cg**	**Sg vs. Cg**
**Down-regulated**	**Fold-change**	**Fold-change**	**Fold-change**
rno-miR-31	0.37^*^	0.42^*^	/
rno-miR-100	0.45^*^	0.40^*^	/
rno-miR-93	0.45^*^	0.48^*^	/
rno-miR-152	0.38^*^	0.40^*^	/
rno-miR-497	0.33^*^	0.44^*^	/
rno-miR-192	/	0.24^*^	0.28^*^
rno-miR-194	/	0.14^*^	0.12^*^
rno-miR-200b	/	0.19^*^	0.27^*^
Up-regulated	Fold-change	Fold-change	Fold-change
rno-miR-122	3.87^*^	2.49^*^	/

*indicates significant difference between the two groups.

/indicates no significant difference between the two groups.

### Real-time PCR analysis validated the results from microarray

**Figure 3:**
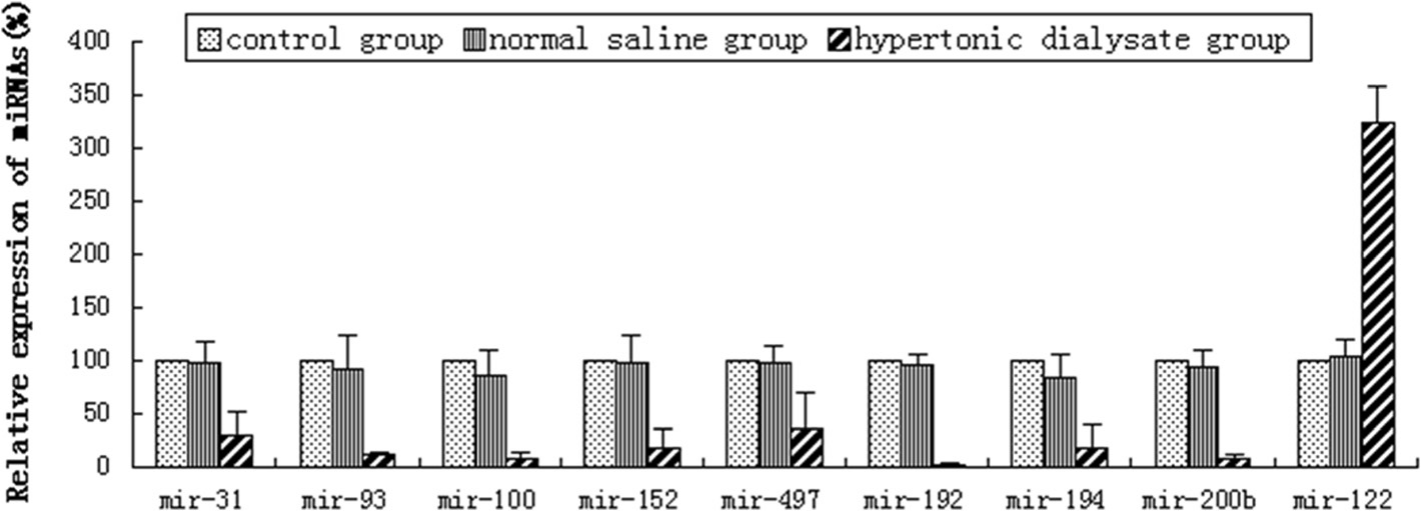
Relative expression of miRNA candidates in three groups after four weeks treatment. Relative expression of miRNAs (%) was measured by real-time PCR analysis and comparisons were made between the control, normal saline and hypertonic dialysate groups. The results are presented as mean ± standard deviation from 3 independent experiments.

Relative expression of the miRNAs identified from microarray analysis was measured by real-time PCR. The results demonstrated that in the hypertonic dialysate group, miR-31, miR-93, miR-100, miR-152, miR-497, miR-192, miR-194 and miR-200b were all significantly down-regulated whereas miR-122 was highly up-regulated (all *P* <0.05) (Figure 3). These results were consistent with those from microarray analysis. However, no significant differences in miRNA expression were observed between the control and normal saline groups (all *P* >0.05) (Figure 3).

## Discussion

In the present study, a rat model of peritoneal fibrosis was successfully established through daily intraperitoneal injection of 4.25% hypertonic dialysate for 4 weeks. This model has been widely used in numerous previous studies and provides valuable information needed to understand the pathological process of peritoneal fibrosis induced by peritoneal dialysis fluid [30,31]. Compared to the peritoneal dialysis performed through abdominal cavity intubation with a catheter, intraperitoneal injection of dialysate has many advantages such as lower cost, higher feasibility and reduced risk of developing exit site infection. In order to assess the potential impact of peritoneal mechanical injury caused by daily injection, a saline group in which the rats received daily peritoneal injection of saline solutions was used and compared to the untreated control group. Our results showed that there were no significant differences in all the indicators of peritoneal fibrosis between saline and untreated control groups, suggesting that the peritoneal mechanical injury was not a confounding factor in our study.

Progression of dialysis-induced peritoneal fibrosis in our rat model led to impaired peritoneal function, which was indicated by reduced ultrafiltration capacity, decreased glucose reabsorption (D_2_/D_0_) and higher dialysate-to-plasma urea ratio (D/P_urea_). During the histological examination, we observed thicker peritoneal membrane and higher collagen deposition. Pathogenesis of peritoneal fibrosis is accompanied by a series of cellular changes including mononuclear cell infiltration, fibroblastic activation, neovascularization and interstitial edema, and all these changes were more prominent in the hypertonic dialysate group. The expression levels of α-SMA, COL-1 and vimentin were all markedly higher in the hypertonic dialysate group, indicating that the peritoneal mesothelial cells had acquired a mesenchymal phenotype through the process of EMT and had enhanced production of extracellular matrix components [36]. In an effort to identify potential regulators of peritoneal fibrosis, a miRNA screen was performed, which represented a parallel and high-throughput method of detecting hundreds of miRNAs simultaneously. Quantitative real-time PCR is the golden standard for gene expression quantification and thus used in our study to validate the results from microarray analysis [37]. Compared with the control and saline groups, both miRNA microarray and real-time PCR analyses demonstrated that miR-31, miR-93, miR-100, miR-152, miR-497, miR-192, miR-194 and miR-200b were significantly down-regulated, and miR-122 was highly up-regulated in the hypertonic dialysate group. Overall, the results from microarray analysis correlated well with those from real-time PCR, which demonstrates the reliability of the miRNA screen and warrants further investigation.

The role of miRNAs in tissue fibrosis has been previously studied in multiple animal models and patients with fibrotic diseases. In a mouse model of pulmonary fibrosis, miR-31 was shown to be down-regulated in the fibroblasts [38]. This result is consistent with ours in the rat model of peritoneal fibrosis. Furthermore, miR-31 was identified as a direct modulator of integrin-α(5) and RhoA, two critical activators of the migratory activity of fibroblasts [38]. These results provide us a clue to find out the targets of miR-31 in peritoneum. In patients with renal fibrosis and IgA nephropathy, the urinary miR-93 level correlated with the degree of glomerular scarring and was regulated by the TGF-β1/SMAD3 pathway [39]. However, miR-93 was found to be up-regulated in patients with renal fibrosis, which is the opposite of our finding in peritoneal fibrosis. The function of miR-93 in tissue fibrosis might be context-dependent and tissue specific. The exact mechanism by which miR-93 regulates the development of progressive renal and peritoneal fibrosis needs further investigation. Decreased expression of miR-192 was detected in patients with established diabetic nephropathy, and its expression level correlated with the degree of tubulointerstitial fibrosis [40]. However, opposite results were shown in a kidney disease model of obstructive nephropathy, in which over-expression of Smad7 inhibited the expression of miR-192 and resulted in suppression of renal fibrosis [41]. Further studies are needed to elucidate how miR-192 exhibits its inhibitory activity on peritoneal fibrosis. miR-194 has been associated with liver fibrosis in a rat model. It was found to be down-regulated in fibrotic liver, similar as we observed in peritoneal fibrosis [42]. Introduction of miR-194 in hepatic stellate cells resulted in significantly reduced expression of α-SMA and COL-1 [42]. These results are consistent with our findings and future studies should focus on elucidating how the targets of miR-194 might be involved with modulation of α-SMA and COL-1 activation. The roles of miR-200 family in EMT and fibrogenesis have been well defined by previous research [43,44]. miR-200a was shown to inhibit EMT and renal fibrosis in a TGF-β-induced diabetic kidney model [45]. Administration of miR-200b could suppress the fibrogenesis of intestinal epithelial cells *in vitro* and ameliorate renal fibrosis in a mouse model of tubulointerstitial fibrosis [46,47]. Taken together, these results suggest to us that the miRNA species identified in our miRNA screen (mir-31, mir-93, mir-192, mir-194 and mir-200b) may be critical in the process of mesothelial cell EMT and in the progression of peritoneal fibrosis. Identification of their molecular targets and exploration of the mechanism by which those downstream molecules coordinate to modulate fibrogenesis deserve further study.

miR-122 is the only miRNA identified to be significantly up-regulated in our rat model of hypertonic dialysate-induced peritoneal fibrosis. As the most abundant miRNA species in hepatocytes, the role of miR-122 in fibrogenesis has been well studied in liver models. The results suggest that miR-122 may contribute to the regulation of tissue remodeling, collagen production and pathogenesis of diet- or hepatitis C virus-induced liver fibrosis [48-50]. Studies of miR-122 in other tissues or disease models are extremely limited. Our result provides additional evidence supporting the role of miR-122 in fibrogenesis.

A recent study identified miR-29b, a downstream inhibitor of TGF-β signaling, as a negative regulator of mouse peritoneal fibrosis [29]. Presence of miR-29b has also been detected specifically in human peritoneal fluid [27]. miR-29b is a member of the miR-29 family which shares the same seed sequence. The role of miR-29 family members in various fibrotic diseases such as renal and liver fibrosis has been reported, and the results indicate that they function through modulation of collagen related gene expression and formation of extracellular matrix [51,52]. The change in expression of miR-29b between control and hypertonic dialysate groups was examined in our microarray analysis. However, no significant difference was observed. The inconsistency might result from the variations in experimental systems, treatment duration, time of assay, and sensitivity of detection methods. Further investigation is required to evaluate the function of miR-29b and other family members in pathogenesis of peritoneal fibrosis.

Although an accumulating number of studies have suggested the role of miRNAs in regulating EMT, only a handful of them focus on peritoneal mesothelial cells [23,53]. EMT involves trans-differentiation of mesothelial cells into fibroblast-like cells and triggers the development and progression of peritoneal fibrosis [25]. Cancer migration and metastasis share a similar mechanism: remodeling of extracellular matrix and initiation of EMT. Several studies have demonstrated that miR-100, miR-152 and miR-497 are associated with the development of tumor and tumor metastasis [54-56]. Thereby, we speculate that above miRNAs may play similar roles in EMT of mesothelial cells and the progression of peritoneal fibrosis. Although the correlation between dysregulated miRNA expression and EMT/peritoneal fibrosis was indicated by the present study, the exact roles these miRNAs play are still unclear. In addition, further studies should be conducted to determine the targets of miRNAs involved in these processes and to elucidate the underlying mechanism. Potential role of the miRNA candidates in other cell types present in the peritoneum, such as vascular endothelial cells and infiltrating mononuclear cells, should also be investigated.

## Conclusions

In the rat model of peritoneal fibrosis established by intraperitoneal injection of 4.25% hypertonic dialysate, a miRNA screen was performed and identified miRNA candidates which might play roles in pathogenesis of peritoneal fibrosis. Strong correlation between altered expression of these miRNA candidates and epithelial-to-mesenchymal transition was also indicated. We believe that a better understanding of the roles these miRNAs play in peritoneal fibrosis will lead to identification of effective diagnostic markers and therapeutic targets in patients with progressive peritoneal fibrosis and ultrafiltration failure during long-term PD.

## Additional files

Additional file 1:
**Sequences of primers used in real-time PCR analysis of mRNA expression.**
Additional file 2:
**Sequences of primers used in real-time PCR analysis of miRNA.**

## Abbreviations

SD: Sprague–Dawley
SPF: Specific pathogen free
miRNA: MicroRNA
PD: Peritoneal dialysis
EMT: Epithelial-to-mesenchymal transition
PDS: Peritoneal dialysis solution
PET: Peritoneal equilibration test
UF: Ultrafiltration
PBS: Phosphate buffered saline
IHC: Immunohistochemistry
HRP: Horseradish peroxidase
SD: Standard deviation
DAB: Diaminobenzidine
AGCC: Affymetrix GeneChip Command Console
α-SMA: alpha-smooth muscle actin
COL-1: type I collagen
