# Supplementary material for: A microrna screen to identify regulators of peritoneal fibrosis in a rat model of peritoneal dialysis
Source: BMC Nephrol. 2015 Apr 9;16:48. doi: 10.1186/s12882-015-0039-z (PMC4546227; doi:10.1186/s12882-015-0039-z)
Supplement: Additional file 1: — Sequences of primers used in real-time PCR analysis of mRNA expression. [file 12882_2015_39_MOESM1_ESM.pdf]

Online Resource 1. Sequences of primers used in real-time PCR analysis of mRNA expression

Gene	Sequences (5' to 3')
GAPDH	GCAAGTTCAACGGCACAG<br>GCCAGTAGACTCCACGACAT
α -SMA	CTTCTATAACGAGCTTCGC<br>TCCAGAGTCCAGCACAAT
COL-1	ACTCAGCCCTCTGTGCCT<br>CCTTCGCTTCCATACTCG
