# Supplementary material for: A microrna screen to identify regulators of peritoneal fibrosis in a rat model of peritoneal dialysis
Source: BMC Nephrol. 2015 Apr 9;16:48. doi: 10.1186/s12882-015-0039-z (PMC4546227; doi:10.1186/s12882-015-0039-z)
Supplement: Additional file 2: — Sequences of primers used in real-time PCR analysis of miRNA. [file 12882_2015_39_MOESM2_ESM.pdf]

Online Resource 2. Sequences of primers used in real-time PCR analysis of miRNA

miRNA	Sequences
miR-31	RT-primer[a]: GTCGTATCCAGTGCAGGGTCCGAGGTA<br>TTCGCACTGGATACGACCAGCTATG<br>F-primer: ATGGTTGGGAGGCAAGATGCTGG<br>R-primer: GGTCCGAGGTATTCGCACT
miR-93	RT-primer: GTCGTATCCAGTGCAGGGTCCGAGG<br>TATTCGCACTGGATACGACCTACCTGC<br>F-primer: ATGGTTGGGCAAAGTGCTGTTCGT<br>R-primer: CGTATCCAGTGCAGGGTCCG
miR-100	RT-primer: GTCGTATCCAGTGCAGGGTCCGAGGT<br>ATTCGCACTGGATACGACCACAAGTT<br>F-primer: ATGGTTGGGAACCCGTAGATCCGAA<br>R-primer: CGTATCCAGTGCAGGGTCCG
miR-152	RT-primer: GTCGTATCCAGTGCAGGGTCCGAGGT<br>ATTCGCACTGGATACGACCCAAGTTC<br>F-primer: ATGGTTGGGTCAGTGCATGACAGA<br>R-primer: TATCCAGTGCAGGGTCCGA
miR-497	RT-primer: GTCGTATCCAGTGCAGGGTCCGAGGT<br>ATTCGCACTGGATACGACTACAAACC

	F-primer: ATGGTTGGGCAGCAGCACACTGT<br>R-primer: GGTCCGAGGTATTCGCACT
miR-192	RT-primer: GTCGTATCCAGTGCAGGGTCCGAGGTA<br>TTCGCACTGGATACGACGGCTGTCA<br>F-primer: ATGGATCGTGGGCTGACCTATGAATTG<br>R-primer: GCAGGGTCCGAGGTATTC
miR-194	RT-primer: GTCGTATCCAGTGCAGGGTCCGAGGT<br>ATTCGCACTGGATACGACTCCACATG<br>F-primer: ATGGTTCGTGGGTGTAACAGCAACTCCA<br>R-primer: GCAGGGTCCGAGGTATTC
miR-200b	RT-primer: GTCGTATCCAGTGCAGGGTCCGAGGT<br>ATTCGCACTGGATACGACGTCATCAT<br>F-primer: TTGGTTCGTGGGTAATACTGCCTGGTA<br>R-primer: GCAGGGTCCGAGGTATTC
U6	RT-primer: CGCTTCACGAATTTGCGTGTCAT<br>F-primer: GCTTCGGCAGCACATATACTAAAAT<br>R-primer: CGCTTCACGAATTTGCGTGTCAT

[a] RT-primer: primer used in reverse transcription PCR
